# Effects of 8-Week Exhausting Deep Knee Flexion Flywheel Training on Persistent Quadriceps Weakness in Well-Trained Athletes Following Anterior Cruciate Ligament Reconstruction

**DOI:** 10.3390/ijerph192013209

**Published:** 2022-10-14

**Authors:** Frederick James Henderson, Yu Konishi, Norihiro Shima, Yohei Shimokochi

**Affiliations:** 1 Department of Health and Sport Management, Osaka University of Health and Sport Sciences, Sennan-gun 590-0496, Japan; 2 Department of Physical Education, National Defense Academy of Japan, Yokosuka 239-8686, Japan; 3 Department of Sport and Health Science, Tokai Gakuen University, Miyoshi 470-0207, Japan

**Keywords:** knee, strength, ACL, inertial training, resistance training, rehabilitation

## Abstract

Persistent quadriceps weakness after anterior cruciate ligament (ACL) reconstruction is a common hurdle to efficient rehabilitation. Therefore, we evaluated a new treatment strategy for athletes with ACL reconstruction. Eleven athletes with unilateral ACL reconstruction performed one set of flywheel Bulgarian split squats to exhaustion with a maximum knee extension of 60°, over 16 sessions, on their reconstructed limb. Quadriceps rate of force development (RFD) 0–50 ms (RFD_0–50 ms_), and 0–150 ms (RFD_0–150 ms_), maximum voluntary isometric contraction (MVIC), and central activation ratio (CAR) were measured bilaterally on the week before and after the intervention. In the reconstructed limb, the RFD_0–50 ms_ (*p* = 0.04; Cohen’s *d* = 0.8) and RFD_0–150 ms_ (*p* = 0.03; *d* = 0.9) increased after training. Before-after changes in MVIC and CAR were not significant (*p* > 0.05), but the lower the baseline MVIC, the greater the gain in MVIC (*r* = −0.71, *p* = 0.02). The between-leg difference in MVIC changed from large before (*p* = 0.01; *d* = 0.8) to small after training (*p* = 0.04; *d* = 0.4). One set of deep knee flexion flywheel Bulgarian split squats to exhaustion improved quadriceps deficits in well-trained athletes with ACL-reconstruction, particularly those with relatively low quadriceps force production.

## 1. Introduction

Persistent quadriceps weakness—a common barrier to rehabilitation after Anterior Cruciate Ligament Reconstruction (ACLR) Ref. [1]—is characterized by a bilateral deficit in muscle activation. Often, this weakness is attributed to a neural mechanism called arthrogenic muscle inhibition (AMI) [2] which possibly stems from incomplete reinnervation of the graft [3,4]. Indeed, the ACL has been suspected to affect gamma motoneurons [5,6], and Konishi et al. [7] confirmed that ACL-deficiency affects the “gamma loop”. ACL tears disrupt knee afferent signaling to γ-motoneurons, thus reducing muscle spindle activity. Group Ia and II afferent signals from muscle spindles to corticospinal structures decline, limiting α-motoneuron activation and quadriceps strength [8]. Consequently, patients are confronted with knee extension deficit, abnormal gait, quadriceps atrophy, poor knee function, dynamic instability, persistent knee pain, or early onset of osteoarthritis [1,2]. Furthermore in athletes, good quadriceps maximum strength, rate of force development (RFD), and activation level also contribute to safe and efficient landing and jumping performance [9,10,11,12]. However, few established treatments are currently available.

Reviewing the efficacy of interventions targeting AMI, Sonnery-Cottet et al. [1] found low- and very low-quality evidence (GRADE score) for electrostimulation, vibration, and ultrasound but moderate-quality evidence for cryotherapy and physical exercise. However, not all forms of exercise efficiently restore quadriceps function. De la Fuente et al. [13] investigated the effects of steady-contraction training coupled with visual feedback over 7 weeks in participants with ACLR who displayed no change in quadriceps strength after 34 weeks of standard rehabilitation. The participants performed five sets of eight 5-s isometric contractions at 70% of their maximum voluntary isometric contraction strength (MVIC) combined with visual feedback to reduce variability in the motor unit (MU) firing rate and increase quadriceps strength. Training successfully increased the knee extensor torque by 60 Nm on average in 84% of the participants. Lepley et al. [14] also reported large gains in quadriceps strength and activation absent from standard care or electrostimulation after eccentric training, in line with reports that eccentric contractions alter cortical activity and down-regulate inhibitory pathways, thereby enabling high-threshold MU recruitment and muscle strength increase [15]. Thus, both studies suggest that exercise that alters the MU firing rate, recruitment threshold, or both may improve quadriceps strength in patients with ACLR.

By involving eccentric and concentric contractions, flywheel training may offer a new way to increase quadriceps muscle activation and reduce ACLR-related quadriceps weakness. A flywheel device uses the moment of inertia rather than gravity for its resistance [16]. A high eccentric muscle load can improve maximal strength [15], and flywheel produces greater eccentric muscle activation than gravity-based training [16]. Importantly, eccentric training is thought to improve strength by reducing neural inhibition through cortical adaptations supported by fusimotor feedback [15], which might reduce gamma loop disruption stemming from an ACL tear. Moreover, the flywheel achieves this greater eccentric stimulus without requiring heavy weights, making it more practical and potentially safer. In addition, higher–threshold MUs are progressively recruited during submaximal muscle contraction carried to exhaustion [17], in line with Henneman’s size principle [18]. Consequently, repeatedly performing sets of resistance training exercise to exhaustion may allow the recruitment of stronger MUs that cannot be activated because of persistent quadriceps weakness. Similarly, quadriceps activation during a single-leg squat is the greatest past 50–60° of knee flexion [19] with little-to-no strain on the ACL at 60° of flexion [20]. Therefore, by limiting the maximum knee extension to 60°, we would not only achieve greater activation of the knee extensors but also do so with marginal ACL graft strain. Therefore, we believe that combining exhaustion with high eccentric and concentric loads achievable on the flywheel during deep knee flexion may facilitate the recruitment of high-threshold MUs to overcome persistent ACLR-related quadriceps weakness.

This approach has several advantages for therapists, strength coaches, and athletes. The flywheel could offer an affordable and practical alternative to expensive and cumbersome equipment used in previous studies, such as isokinetic dynamometer [13], computerized leg press [14], and magnetic and electrical stimulators [1]. By limiting the intervention to a single set carried to exhaustion, this ACLR-specific training would be time–efficient and interfere little with the athletes’ regular training, as the training load is relatively small. In addition, RFD affects athletic performance [21], involves neural function [22,23], is affected by ACL injuries [21,24], and can improve with training [24,25]. However, quadriceps RFD measures appear to be absent from previous studies. Therefore, clarifying the effects of flywheel training on quadriceps AMI is of clinical benefit.

Hence, we hypothesized that a single set of deep knee flexion Bulgarian split squats to exhaustion of the reconstructed limb would significantly improve quadriceps RFD, maximal strength, and voluntary activation.

## 2. Materials and Methods

### 2.1. Design

In this non-randomized intervention study in athletes with ACLR, we measured the RFD from 0 to 50 ms (RFD_0–50 ms_) and from 0 to 150 ms (RFD_0–150 ms_), MVIC, and the central activation ratio (CAR) in the reconstructed and uninvolved legs, before and after 8 weeks (16 sessions) of the flywheel Bulgarian split squat, limiting maximum knee extension at 60° (0° = full knee extension) on the reconstructed leg only.

### 2.2. Subjects

Eleven collegiate athletes with unilateral ACLR were recruited from among the sports clubs of Osaka University of Health and Sport Sciences (descriptive data in Table 1). We recruited at least 10 participants to achieve a statistical power of 0.90 at an alpha level of 0.05, based on a previously reported large-effect size [14] using the GPower software (GPower, version 3.1.9.4, Franz Faul, Universität Kiel, Germany). Inclusion criteria were (i) unilateral ACL injury, (ii) being at least 6 months post-surgery, (iii) having completed rehabilitation at the hospital under the supervision of a therapist, and (iv) having received medical clearance for unrestricted sports participation. All but one of the participants practiced resistance training at least five times a week. Participants provided written informed consent, received detailed explanations, were free to ask questions anytime, and could cease the experiment anytime without justification.

### 2.3. Flywheel Training

**Figure 1:**
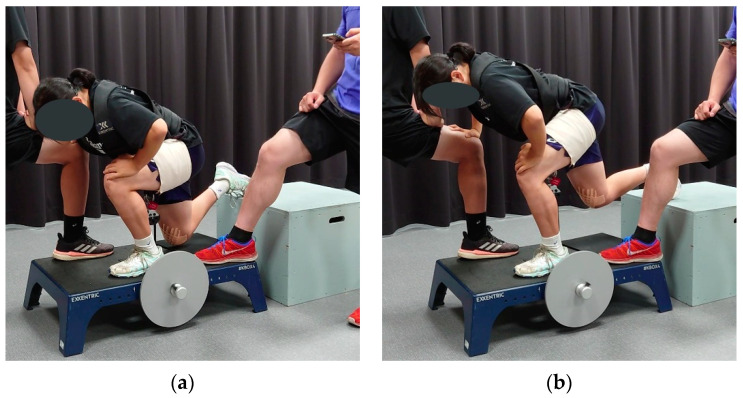
Setup for the Bulgarian split squat on the flywheel. (**a**) Starting/lowest position; (**b**) Highest position.

Participants performed one set of Bulgarian split squats (see Figure 1) to exhaustion (participants could do no additional repetition) on the reconstructed leg using a flywheel (KBOX4, Exxentric AB, Bromma, Sweden), twice a week for eight weeks, at least 48 h apart, in addition to their regular training. The uninvolved leg did not undergo specific training. The length of the flywheel strap was set such that the maximum knee extension was 60° of knee flexion (0°: full extension; Figure 1b). The knee angle was defined as the angle between the line connecting the lateral malleolus to the lateral femoral epicondyle and the line connecting the lateral femoral epicondyle to the greater trochanter, measured with a handheld goniometer. The starting position is illustrated in Figure 1a: ankle aligned with the wheel axis, front leg parallel to the ground with the knee below the tip of the toes, leading to a knee angle of approximately 100°. The effective range of motion was thus 100–60° for all participants. The participants were instructed to gently touch an 8 mm–thick foam pad on the flywheel platform with the suspended knee. Participants could rest one hand on the knee of the experimenter in front of them for balance and safety. The initial moment of inertia was 0.025 kg.m^2^ for female and 0.050 kg.m^2^ for male participants. The moment of inertia was increased in the following session by 0.025 kg.m^2^ once a participant could realize 20 “strong” repetitions (repetitions achieving an average power over 80% of the averaged three highest-powered repetitions). Training data were recorded using the flywheel manufacturer’s mobile application (Exxentric kMeter 3.9.1(3) for iOS 15.4.1). Encouragement and feedback were provided during training. Each participant was trained individually at the laboratory, training room, or handball court—as per the participant’s preference. The author (FH), a Master’s in Sports Science and Professional Strength coach for over 15 years, supervised the training and collected data.

### 2.4. Quadriceps Measure Setup

Participants sat on a custom-made leg extension chair used previously [9], with hips flexed at approximately 70° (0°: trunk and femur aligned) and 90° of knee flexion. The leg was strapped to a force transducer (TSA-110, Takei Scientific Instruments Co., Ltd., Niigata City, Japan), with the hips and chest belted to the seat. The force transducer was linked to an analog interface (PowerLab/8sp, ADInstruments Pty, Ltd., Castle Hill, Australia), and data were collected via software (LabChart 6, ADInstruments NZ Limited, Dunedin, New Zealand) at a sampling rate of 1 kHz.

Two electrode pads were strapped to the quadriceps: 20 cm × 10 cm on the proximal part (middle of the upper third of the thigh) and 20 cm × 10 cm for male or 15 cm × 10 cm for female participants on the distal part (middle of the lower third of the thigh), to fully cover the quadriceps muscle belly without covering the knee flexors. The electrodes were connected to a high–voltage, constant–current stimulator (DS7AH, Digitimer, Hertfordshire, UK) linked to the same analog interface as the transducer and controlled via the same software.

### 2.5. Electrostimulation Calibration & Warm-up

**Figure 2:**
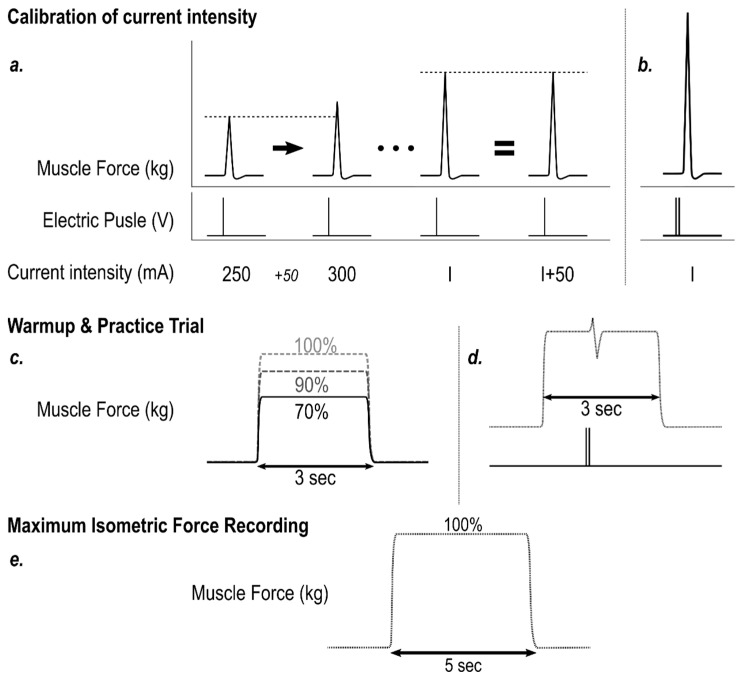
Calibration and warmup procedure for the superimposed twitch technique. (**a**). Starting at 250 mA, a single pulse is triggered, and force is recorded. Amperage is increased in 50 mA increments until force plateaus. All motor units are now considered recruited. The before-last amperage (I) is used for the experiment. (**b**). The actual trial employs double twitch. Tolerance to pain and discomfort is confirmed by the participant. (**c**). Warm up trials at the participant’s perceived 70%, 90%, and 100%. (**d**). Practice trial. (**e**). 5-s maximum isometric voluntary contraction. The highest 3-s average is extracted. The threshold for valid trials is 90% of this 3-s maximum force.

Electrical pulse frequency was set to 100 Hz, amplitude to 5.5 V, and width to 0.5 ms. The maximal current intensity was determined individually by stimulating the resting quadriceps with single twitches and recording the torque output in a stepwise fashion, starting from 250 mA and increasing the current in increments of 50 mA, until the torque did not increase anymore, at which point all motor units were considered to be recruited [26]. Double-stimulation twitches were used during measurement to ensure full muscle activation. Previous pilot experimentation confirmed that full quadriceps activation was achieved using this procedure and was generally well tolerated by the participants. The warm-up consisted of three 3-s MVICs at 70, 90, and 100% of the participant’s perceived maximum strength, one practice trial with electrostimulation, and a 5-s MVIC. The pre-trial procedure is shown in Figure 2. The uninvolved leg was measured first.

### 2.6. MVIC

The MVIC trials were valid if the force plateau reached 90% of the maximum strength average (Figure 2e) [23]. Three valid trials with electrostimulation and three without electrostimulation (should discomfort or anticipatory stress from electrostimulations reduce voluntary drive) were collected alternatively, with a 30-s rest in-between. The participants decided whether to start the series of trials with or without electrostimulation. Before each MVIC, the participants were instructed to extend their knee as fast and as hard as possible on a vocal signal and exert maximum effort for 3 s. For trials with electrostimulation, the experimenter triggered electrostimulation once the force visibly plateaued. The participants received encouragement and were cheered during each trial.

### 2.7. RFD and MVIC

RFD_0–50 ms_, RFD_0–150 ms_, and MVIC were extracted from each trial, and the three highest values were averaged for analysis. Force measurements during explosive isometric contractions have proven reliable, with good–to–excellent intraclass correlation for force production of 0.80 at 50 ms, 0.90 at 150 ms, and 0.95 for MVIC [27].The lever arm was measured between the femur lateral condyle and middle of the ankle strap to convert force measures to torque. Data were normalized to the participant’s body weight. Contraction onset was determined visually as previously advocated [28]. Data were filtered using a low-pass filter at a cut-off frequency of 12 Hz before analysis. The MVIC was the highest value recorded during the trial or before electrostimulation.

### 2.8. Central Activation Ratio

According to previous literature [29], CAR was defined as:

$$\mathrm{CAR}=\frac{\mathrm{MVIC}}{\mathrm{MVIC}+\mathrm{superimposed}\;\mathrm{twitch}\;\mathrm{torque}}\times 100$$

where MVIC is the force 1 ms before the electrostimulation. We retained the highest recorded CAR for the analysis [30]. CAR has proven reliable with an intraclass correlation of 0.86—0.94 [31].

### 2.9. Analysis

We conducted pre-planned, one-tailed, paired t-tests for pre–post measures because we hypothesized that the measured variables would increase after intervention. The alpha level was 0.05. Additionally, we performed two-way, mixed-design ANOVAs with time as a within-subjects and legs as a between-subjects factor comparison. The alpha level for ANOVAs was 0.10 because 2-way ANOVAs are two-tailed, unlike our hypothesis. Post–hoc one-tailed, paired t-tests were performed for significant outcomes. Friedman and Wilcoxon matched-pairs signed rank tests were substituted for variables that were not normally distributed. We evaluated normality using the Shapiro–Wilk test at an alpha level of 0.05. Cohen’s *d* was calculated for significant differences with d(0.1) = trivial, d(0.2) = small, d(0.5) = medium, and d(0.8) = large effect [32]. Per Cohen still [32], effect size expressed as partial η^2^ for ANOVA yielded η^2^(0.00) = trivial, η^2^(0.01) = small, η^2^(0.06) = medium, and η^2^(0.14) = large effect. Relationships between variables’ baseline value and percentage of change were evaluated a posteriori using Pearson’s *r* with r(0.1) = small, r(0.3) = moderate, and r(0.5) = large effect [32]. Analysis was performed using the RStudio software (RStudio, version 2022.2.3.492, PBC, Boston, MA, USA).

## 3. Results

### 3.1. Execution of the Experiment

Every participant completed every session without adverse events and showed an improvement in the average power and number of repetitions over the 16 sessions. Two subjects did not tolerate electrostimulation well but managed at least one valid electrostimulation trial that was analyzed. In one participant, electrostimulation equipment failure did not allow for proper calibration. Consequently, the CAR for this participant could not be recorded, and only 10 subjects were included in the CAR analysis. One subject complained of light quadriceps pain in the uninvolved leg on the post-measurement day. The session was rescheduled once the pain had subsided (48 h later). All the participants completed three valid trials without electrostimulation.

### 3.2. Pre-post Comparisons

**Figure 3:**
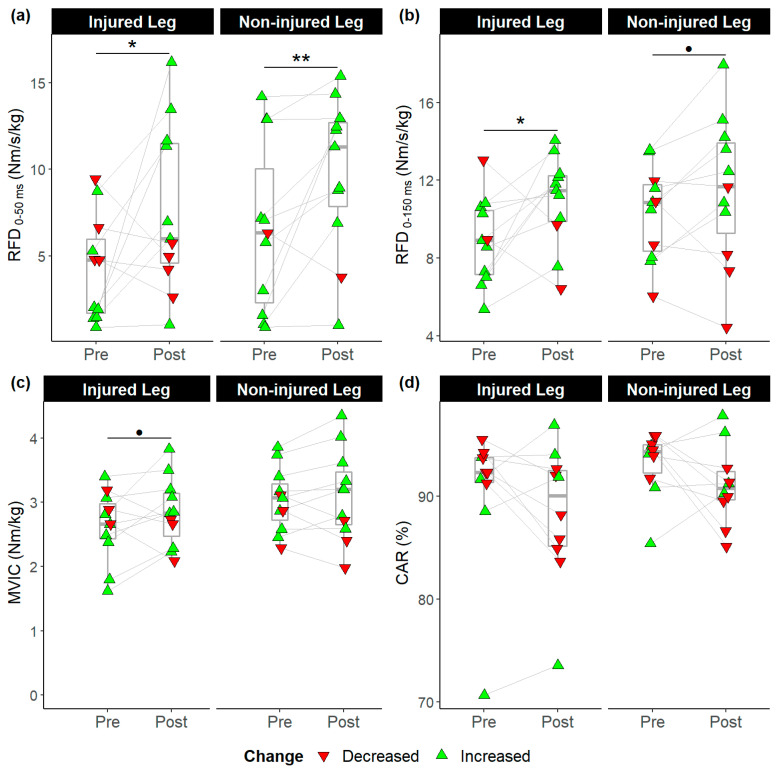
Pre- and post-intervention measures. (**a**) Rate of force development from 0 to 50 ms (RFD_0–50 ms_); (**b**) Rate of force development from 0 to 150 ms (RFD_0–150 ms_); (**c**) Maximum voluntary isometric contraction strength (MVIC), (**d**) Central activation ratio (CAR); ●: *p* ≤ 0.10, *: *p* ≤ 0.05, **: *p* ≤ 0.01. Green triangles indicate participants who improved after intervention; red triangles indicate participants who did not.

The Shapiro–Wilk test confirmed that CAR data before the intervention were not normally distributed (*p* < 0.01), while all other variables were normally distributed (*p* = 0.15–0.99). RFD_0–50 ms_ increased from (mean ± SD) 4.31 ± 3.03 to 7.66 ± 4.80 Nm/s/kg in the reconstructed leg (*p* = 0.04, *d* = 0.8), and from 6.63 ± 4.89 to 9.83 ± 4.48 Nm/s/kg in the uninvolved leg (*p* = 0.01, *d* = 0.7). RFD_0–150 ms_ increased from 8.87 ± 2.23 to 10.94 ± 2.34 Nm/s/kg in the reconstructed leg (*p* = 0.03, *d* = 0.9). The other changes were not significant (Figure 3).

### 3.3. Between-Leg Comparisons

A two-way ANOVA revealed no significant time × leg interaction effect for any variable (*p* = 0.59–0.95, *η*^2^ = 0.00–0.01). A possible effect of leg reconstruction on MVIC was observed (*F*(1,40) = 3.71, *p* = 0.06) and was further investigated (Table 2).

### 3.4. Baseline—Change Amplitude Correlation Anaylsis

**Figure 4:**
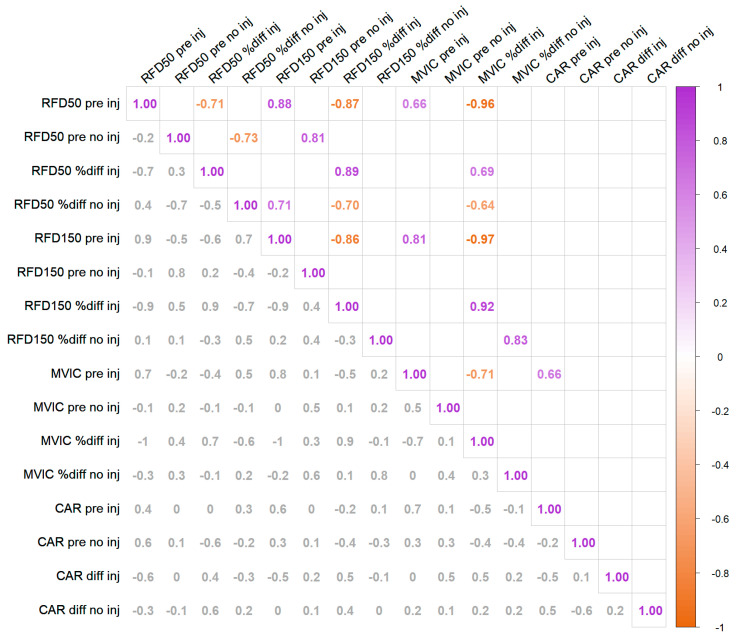
Correlation matrix plot between baseline measure and magnitude of change induced by the intervention. The upper-right part of the matrix only displays significant correlations, with *p* ≤ 0.05. The bottom left shows all the correlation coefficients regardless of significance, for reference. CAR data from participant #11 were missing and were excluded from this analysis, therefore only data from 10 subjects are included. RFD: Rate of force development 0–50 ms (RFD50) and 0–150 ms (RFD150); MVIC: Maximum voluntary isometric contraction force; CAR: Central activation ratio; pre: measure pre-intervention; %diff: percentage of change between pre and post intervention measurements; inj: injured leg; no inj: non-injured leg.

Baseline RFD_0–50 ms_ was strongly and negatively associated with the percent change in RFD_0–50 ms_ (*r* = −0.71, *p* = 0.02), RFD_0–150 ms_ (*r* = −0.87, *p* = 0.00), and MVIC (*r* = −0.96, *p* = 0.00). Similarly, RFD_0–150 ms_ was associated with changes in RFD_0–150 ms_ (*r* = −0.86, *p* = 0.00) and MVIC (*r* = −0.97, *p* = 0.00). MVIC was also associated with a change in MVIC (*r* = −0.71, *p* = 0.02) (Figure 4).

## 4. Discussion

In this study, we tested the hypothesis that one set to exhaustion of deep knee flexion Bulgarian split squats on the reconstructed leg using a flywheel device would significantly improve MVIC, RFD_0–50 ms_, RFD_0–150 ms_, and CAR in well-trained athletes who completed standard rehabilitation. RFD_0–50 ms_ and RFD_0–150 ms_ significantly increased, but CAR did not. Although the gains in MVIC were not significant, correlation analysis revealed that the lower the baseline MVIC, the greater the gain in MVIC. Our findings suggest that the one set to exhaustion of deep knee flexion in Bulgarian split squat flywheel training can improve quadriceps function in well-trained athletes with ACLR, but the effect on quadriceps strength may be greater in individuals with weaker quadriceps.

The most notable results of our study were how RFD measures and CAR changed after our intervention, reflecting changes in neural function. Quadriceps electromyographic activity is the strongest determinant of explosive force throughout 0–150 ms of forceful knee extension [22], particularly prior to 75 ms [22,33]. Thus, changes in RFD_0–50 ms_ and RFD_0–150 ms_ partly reflect changes in MU firing rate. In parallel, CAR would reflect how much of the MU pool could be voluntarily recruited by the participant.

Quadriceps RFD_0–50 ms_ and RFD_0__–150 ms_ in the reconstructed limb showed significantly large improvements (30.6% and 20.6%, respectively). Gains in RFD were not homogeneous though, as only 6 of 11 participants showed great RFD_0–50 ns_ but 9 of 11 showed improvements in RFD_0–150 ms_. The negative correlation between the baseline value and subsequent increase was large for RFD_0–50 ms_ (*r* = −0.71, *p* = 0.02) and RFD_0–150 ms_ (*r* = −0.86, *p* = 0.00). We found no comparable measures in the ACLR population; however, Folland et al. [22] used similar variables among healthy participants, reporting mean MVIC, force at 50 ms, and force at 150 ms of 459 N, 83 N, and 347 N, respectively. In comparison, the mean end-values were 553 N, 75 N, and 317 N, respectively, in this study. Differences in measuring methods notwithstanding, our end-values appear close to those of healthy participants, suggesting that our intervention helped restore early force production. These gains are all the more important because RFD is crucial to athletes; many sports movements are rapid, requiring 50 to 200 ms, but the time required to reach maximal muscle force is estimated to be 300 ms [34] For example, ground contact time in sprinting has been clocked at less than 100 ms [35].

On the contrary, the baseline CAR in our sample averaged 90.4% (range: 70.69–95.53%) and did not significantly increase after training. While mean CAR was several percent below the values in Lepley et al. [14], 9 out of 10 participants had a CAR above 88.5%, which is within the normal 85–95% range for healthy participants [36], including elite athletes [37]. Therefore, our participants possibly had already recovered normal quadriceps activation, and the intervention’s training stimulus may have been insufficient to further improve maximal MU recruitment. Indeed, training–induced increase in CAR is not systematic [36], and neural adaptations develop more slowly in chronically trained athletes like ours [38]. Alternatively, changes in muscle architecture, such as muscle hypertrophy, may also increase MVIC at the same activation level. Consequently, the hypothesis that exhaustive deep knee flexion training increases quadriceps activation during MVIC in athletes with ACLR is not supported.

Taken together, while unchanged CAR suggests that more MUs were not recruited, gains in RFD suggest that flywheel training altered MU behavior. Thus, we conclude that exhaustive deep knee flexion flywheel training can improve neural drive during early force production in athletes with ACLR. Eight of the eleven athletes with ACLR showed improvement (range: 3.9–27.4%), including five individuals showing gains beyond 20% (range: 21.4–27.4%), although pre–post comparison showed that MVIC in the reconstructed leg did not significantly increase (*p* = 0.09, *d* = 0.4 [small]). Furthermore, the interlimb difference improved from significantly large to small (Table 2). Correlation analysis between baseline MVIC and percentage of MVIC change showed that the lower the baseline, the greater the improvement (*r* = −0.71, *p* = 0.02). Collectively, these results indicate that most participants showed improved MVIC, but the training effect depended on baseline MVIC. This pattern is reminiscent of the changes in squat 1 RM among professional rugby players observed by Appleby et al. [39], where the higher the baseline strength, the smaller the gains over time. In the study by Lepley et al. [14], who measured MVIC similarly to us, the eccentric-only group achieved an MVIC of 2.80 Nm/kg at the time of return to sport (2.10 Nm/kg upon completing a 6-week eccentric training intervention shortly after ACLR). In our study, MVIC increased on average from 2.56 to 2.77 Nm/kg. Consequently, our sample was already near return-to-sport values and may have had less room to improve on account of being further along in their recovery and training regularly. Thus, it may be that our sample size provides insufficient power to detect a small-effect size. Based on these results, we conclude that exhaustive, deep knee flexion flywheel training increased quadriceps MVIC in athletes with ACLR; however, athletes with ACLR with relatively strong quadriceps may need different training to further improve MVIC.

Finally, our intervention has clinical relevance for preventing ACL reinjuries after returning to sports. We restricted the maximum knee extension to 60° to specifically improve knee-extensor strength and endurance at deeper knee flexion angles. Performing sudden cutting or landing motions with a shallow knee flexion angle is known to contribute to ACL injuries [40]. In contrast, lowering the body’s center of mass through deeper knee flexion contributes to safely changing directions during cutting [41] or jump landing [42,43]. However, knee flexion beyond 60° is more challenging in weight-bearing motions because the ability to produce knee extension moment decreases beyond 60° of knee flexion [44], whereas external knee flexion loading increases along the knee flexion angle [45]. Thus, improving quadriceps function at deeper knee flexion angles should contribute to a safe return to sports while improving athletes’ performance after ACLR. Additionally, the Bulgarian squat on the flywheel also has several practical advantages for therapists and trainers: (i) athletes get accustomed to inertial training rapidly in two to three sessions [46]; (ii) it is a common exercise in training programs, meaning that this movement requires little learning and can be implemented even by trainers with little experience; (iii) it requires little instruction from the trainer to be executed properly, besides reminding athletes to gently touch the platform for each repetition; and (iv) setup of the equipment is quick, simple, and only requires a goniometer to set the maximum knee angle to 60°. Lastly, this protocol proved safe with no adverse events recorded, and no pain but delayed onset muscle soreness the day following the first couple of sessions was reported.

Similar to all other studies, our study has limitations. The study population consisted of well-trained athletes with ACLR. Consequently, our results could apply to similar ACLR populations, such as competitive athletes, military and law enforcement personnel, and firefighters. In addition, while the number of participants was sufficient to detect large or possibly moderate-effect sizes, it was insufficient to directly detect smaller effects, as was the case for MVIC. The time from surgery ranges from approximately 9 months to 2 years for most participants which may have influenced our results. Sex was not uniform, but sex has been shown not to influence explosive strength [22] or CAR [47], and normalizing MVIC by bodyweight will have limited sex differences in strength [48]. While we used the uninvolved leg as a control, we could not control for individual training programs outside of the intervention. Consequently, we cannot conclude whether the bilateral effect in RFD_0–50 ms_ is solely due to our intervention or non-flywheel training. However, the effect was greater in the reconstructed leg; therefore, we can attribute changes in the reconstructed leg mostly to the intervention.

## 5. Conclusions

Although athletes with ACLR may display near–normal quadriceps MVIC, they can still largely improve RFD, suggesting persistent deficits in force production. In practice, therapists should be mindful of RFD restoration along the MVIC as it may impact subsequent athletic performance. In addition, performance coaches should be aware that, despite receiving medical clearance for unrestricted sports participation, athletes with ACLR may need additional quadriceps-specific training. Fortunately, simply adding a single set of Bulgarian split squat on a flywheel device twice a week over a couple of months to an individual’s training program appears sufficient to progressively improve quadriceps force production in athletes who underwent ACLR, particularly in participants with relatively low quadriceps force production.

## Figures and Tables

**Table 1:** Participant information. Dom. Leg: Dominant leg, take-off leg for one-legged jump; BPTB: Bone-patellar tendon-bone; SD: Standard deviation. Days from surgery are counted until the day of pre-intervention measure.

Subject	Height (m)	Weight (kg)	Age (Years)	Dom. Leg	Injured Leg	Graft	Sport	Sex	Days from Surgery
1	1.72	60.3	21	Left	Left	Hamstrings	Handball	Female	405
2	1.75	93.1	22	Left	Left	Quadriceps	Handball	Male	858
3	1.63	63.5	21	Left	Right	Hamstrings	Handball	Female	354
4	1.61	57.8	19	Left	Left	Hamstrings	Handball	Female	374
5	1.64	58.3	20	Right	Right	Hamstrings	Handball	Female	346
6	1.71	66.1	20	Left	Left	Hamstrings	Soccer	Male	288
7	1.63	57.0	19	Left	Left	BPTB	Volleyball	Female	343
8	1.73	76.5	19	Left	Right	Hamstrings	Handball	Female	1053
9	1.83	66.6	28	Left	Right	Hamstrings	Volleyball	Female	783
10	1.86	73.5	19	Left	Left	Hamstrings	Basketball	Male	657
11	1.59	58.6	19	Right	Left	Hamstrings	Handball	Female	589
Mean	1.71	67.3	20.8						546.1
SD	0.09	11.2	2.7						270.2

**Table 2:** Summary of the one-tail, paired t-test statistics and effect size. RFD: Rate of force development (Nm/kg/s); MVIC: Maximum voluntary isometric contraction (Nm/kg); ACLR: ACL-reconstructed limb; *t*: t-value; *df*: Degrees of freedom; *p*: *p*-value; MD: Mean difference; SD: Standard deviation; 95%CI: 95% Confidence interval.

KERRYPNX		*t*	*df*	*p*	MD (SD)	95%CI	Cohen’s *d*	Magnitude
RFD_0–50 ms_	ACLR *	1.98	10	0.04	3.35 (5.61)	[0.28, +∞)	0.8 [−0.2, 1.9]	Large
Pre-Post	Uninvolved *	2.60	10	0.01	3.20 (4.08)	[0.97, +∞)	0.7 [0.1, 1.3]	Medium
RFD_0–150 ms_	ACLR*	2.15	10	0.03	2.08 (3.20)	[0.33, +∞)	0.9 [−0.1, 2.0]	Large
Pre-Post	Uninvolved	1.57	10	0.07	1.15 (2.44)	[−0.18, +∞)	0.3 [−0.1, 0.7]	Small
MVIC	ACLR	1.42	10	0.09	0.21 (0.49)	[−0.06, +∞)	0.4 [−0.2, 1.0]	Small
Pre-Post	Uninvolved	0.74	10	0.24	0.07 (0.32)	[−0.10, +∞)	0.1 [−0.2, 0.4]	Trivial
MVIC	Pre *	2.53	10	0.01	0.40 (0.53)	[0.12, +∞)	0.8 [0.1, 1.5]	Large
Interlimb	Post	1.92	10	0.04	0.27 (0.46)	[0.01, +∞)	0.4 [−0.1, 0.9]	Small

*: *p* ≤ 0.05.

## Data Availability

Data is available upon reasonable request to the corresponding author.
